# Characterization of recruitment through tandem running in an Indian queenless ant *Diacamma indicum*

**DOI:** 10.1098/rsos.160476

**Published:** 2017-01-18

**Authors:** Rajbir Kaur, Joby Joseph, Karunakaran Anoop, Annagiri Sumana

**Affiliations:** 1 Behaviour and Ecology Lab, Department of Biological Sciences, Indian Institute of Science Education and Research, Kolkata, Mohanpur 741246, India; 2 Institute of Zoology, Johannes Gutenberg University of Mainz, Johannes von Müller Weg 6, 55099 Mainz, Germany; 3 Center for Neural and Cognitive Sciences, University of Hyderabad, Hyderabad 500046, India

**Keywords:** *Diacamma indicum*, communication, direct recruitment, path efficiency, tandem running speed

## Abstract

Tandem running is a primitive recruitment method employed by many ant genera. This study characterizes this behaviour during the recruitment of colony mates to a new nest in an Indian ant *Diacamma indicum*. Tandem leaders who have knowledge of the new nest lead a single follower at a time, to the destination by maintaining physical contact. In order to characterize tandem running, we captured and analysed 621 invitations, 217 paths and 226 termination events. Remarkably, not a single colony member was lost. While invitations were stereotypic in behaviour, termination was not. Analysis of speed revealed that the average transport speed was 4.2 cm s^−1^. Coupled adult-brood transport was slower than other transports but was more efficient than individual trips. Comparing tandem running with other popular recruitment methods in ants allows us to postulate that even though tandem running is primitive it is probably just another means to achieve the same end.

## 1. Background

Social insects live in groups ranging from a few to over millions of individuals within a nest. In order to coordinate their activities, communication between members of the colony becomes important. The communication system employed to transfer information within their colony ranges from visual, acoustic, tactile, magnetic and chemical signals [1]. Routine tasks in these insect societies like foraging and nest defence necessitate recruitment of members. One such task that requires the recruitment of not just a few adults, but all the adults of the colony, is relocation. The entire colony needs to be moved to a new location without loss of colony cohesion. Relocation frequency varies from species to species based on their life-history traits and ecological conditions [2,3]. Irrespective of how frequently this event occurs, safe transport of the colony members and any stored resources, while maintaining colony cohesion would be essential. The dynamics of colony relocation with regard to certain important aspects like site discovery, nest-site selection, information transfer to nest-mates and final shift of colony members has been addressed in some social insects [4]. Unlike honeybees and wasps, relocation in ant colonies involves the transport of brood in addition to adult nest-mates, increasing the complexity of the process.

Recruitment methods employed by different ant species include mass recruitment by pheromone trail, direct recruitment by tandem running and transport by carrying. Unlike mass recruitment, direct recruitment requires more time and energy from scouts. Details on relocation dynamics have been documented in few ant species, namely, *Temnothorax albipennis* [5]*, T. curvispinosus* [6]*, Aphaenogaster senilis* [7] and *Diacamma indicum* [8]. This study focuses on tandem running, a direct recruitment method employed by many species of ants across different subfamilies to recruit colony members to available food source, towards new nest site or during slave raids [2,9,10].

Tandem running was first observed by Adlerz in 1896 as documented by Hingston and Stuart [10]. This information was consolidated and further clarified in 1959 by E. O. Wilson [11]. In brief, it is a behaviour involving two individuals walking one behind the other in tandem, maintaining physical contact. The individual in front has prior knowledge of the destination, be it the new nest or food source or nest of slave species, and is known as tandem leader. The follower or second individual in the pair is the recruit and she is led to the destination. The recruited member in turn can behave as an informed individual and recruit other colony members or be only a follower and stay at the destination. Throughout the journey, the follower maintains contact by tapping her antennae on the gaster of the tandem leader thereby forming a tandem running pair [11]. In some species of ants, pheromones are said to play an important role in initiating and maintaining cohesion between the tandem pair [12–14]. Studies in *T. albipennis* suggest that followers learn the destination and make independent explorations to navigate back to the old nest and become recruiters in turn [15,16].

When the cohesion between the leader and follower is lost, tandem runs get interrupted. Studies on *Temnothorax* spp. found that about three-quarters of all tandem runs were interrupted before reaching the destination [6,17]. When interrupted, both tandem leader and follower undertake a systematic search and if reunited they head towards the destination. These tandem run interruptions have been interpreted as teaching [15]. It is argued that tandem leaders (TL) modify their behaviour in order to give their followers an opportunity to learn the path during interruptions. In *T. albipennis* and *T. curvispinosus*, the only other species studied in this context, it is believed that TL search for their follower based on the consideration of location and quality of follower [18]. However, in some cases followers of interrupted tandem runs are known to discover the new nest by extrapolating the path [19,20]. This is not very surprising as followers in these two species are mostly scouts who are expected to be capable of navigating the arena. Among ants, the most common mechanism of recruitment is pheromone trails [2]. An odour trail laid on the ground is used to inform colony members the path from their nest to the destination. In some species, scouts are known to make multiple trips along the trail and influence the movement of colony members [7]. Besides chemical trails and tandem running the third known mechanism of recruitment in ants is carrying. In carrying, the transporter lifts a colony member from the old nest and walks all the way to the new nest to drop them. Carrying is also employed in combination with various other modes of transport [2]. For example *T. albipennis* and *T. curvispinosus* employ tandem running at the beginning of the relocation process and later switch to carrying [5,21]. A study on *T. curvispinosus* found that switching from tandem running to carrying increases the overall efficiency of colony relocation [19].

Unlike these *Temnothorax* spp., *D. indicum* employs only one recruitment method throughout the relocation process [8,22,23]. In this study, our main aim was to characterize tandem running behaviour in *D. indicum*. We carried out this aim by subdividing this behaviour into three sections in the sequence of their occurrence. We started by investigating invitation calls to fellow colony members, i.e. initiation, and then we investigated the manner in which tandem running progresses, i.e. path, and finally, the manner in which the tandem pair end their tandem run, i.e. termination. In addition, we calculated the speed and path efficiency of tandem running in different contexts during relocation and place tandem running in perspective by contrasting it with other recruitment methods in ants.

## 2. Material and methods

Six colonies of *D. indicum* were collected from their natural habitat in Mohanpur (Nadia district, West Bengal, India, 22°56′ N, 88°31′ E) during May 2013 by employing the water stress method [8]. The colonies consisted of 116.2 ± 45.3 adult females, 43.8 ± 15.9 pupae, 32.8 ± 20.3 larvae and 90.0 ± 13.0 eggs. Each ant in a colony was uniquely marked with enamel paint colours (Testors, Rockford, IL, USA) and colonies were housed inside the laboratory where they were provided ad libitum ant food [24], water and termites occasionally. A single relocation was performed with each colony in the experimental arena with dimension 0.9 m (length) × 0.6 m (breadth). Relocation was induced by lifting the roof of the old nest (an opaque watch glass). At the same time, to increase luminosity, temperature and induce air circulation inside the nest, an incandescent light bulb and ceiling fan were turned on. The distance of the nest from light source and fan measured 20 cm and 2.5 m, respectively. Relocation is the process by which organisms move from their old nest to a new one along with all the inhabitants. In our experiments, the distance from the old to the new nest was approximately 1 m, which is comparable to the average distance across which relocations occur in nature [8]. Transport events were recorded at 30 fps using three video cameras (Sony handycam model HDR-CX200) in order to capture: initiation, path and termination of tandem runs. Old nest containing the colony was placed at a random corner of the arena and a video camera placed above it in order to record the initiation of tandem runs. Another video camera was set-up above the arena in order to characterize the path taken by leaders as they tandem ran colony members. This camera was placed at 1.2 m from the sand base of the arena in order to cover the entire arena; however, these recordings were not amicable to deciphering the individual identities of leaders and followers. A third camera was placed above the new nest in order to record the termination behaviour. The new nest consisted of a tunnel-chamber structure, mimicking the natural architecture of *D. indicum* nest (A.S. 2009, personal observation). The tunnel consisted of a transparent plastic tube (15 cm long and 2 cm in diameter) attached to a transparent plastic box measuring 7.2 × 4.8 × 2.5 cm which acted as the nest chamber. Video data were decoded and analysed separately for each of the events, i.e. initiation, path and termination. In the case of initiation and termination, the activities of individual transporters were decoded.

The different categories used to characterize movement between old and new nest are defined below. TL: individuals who lead tandem runs. Followers (F): colony members who follow tandem leader to reach the new nest. Tandem run with brood (TRB): those tandem runs where the follower carries a pupa or a large larva in her mandibles while walking in tandem with a tandem leader to reach the new nest. Tandem run without brood (TR): tandem runs in which a follower who is not carrying any pupa or large larva walks in tandem with a tandem leader to reach the new nest. Brood transport (BT): transport events where a transporter carries a pupa or large larva and walks to the new nest. During BT, TL or ants who have not led any tandem runs either picked brood item that was lying on the ground of old nest or snatched it from other colony members and walked to the new nest. As reliable documentation of transport of eggs and small larvae were not possible due to their small size, they were not included in BT or TRB.

### 2.1. Initiation

In order to initiate a tandem run, TL seem to perform a distinct behaviour termed invitation call to potential followers (electronic supplementary material, media S1). This invitation call has been described as a jerky movement performed by TL and includes pulling of the antenna, leg or body of the potential follower [2]. *Diacamma indicum* invitation was qualitatively similar to what was described in *Camponotus sericeus* species [12]. In order to quantify the invitation behaviour the following protocol was used. For every invitation call the identity of tandem leader, number of jerks (henceforth termed as calls), number of individuals towards whom these calls were made and invitation duration of a given bout of calling and its success or failure was recorded. If tandem leader found a follower and initiated tandem run, it was scored as a successful call, while a failure was recorded if tandem leader stopped calling and walked out of the recording area, i.e. old nest. Owing to large size of one of the colonies, the initiation site became very crowded. This crowding imposed partial difficulties in delineating the number of calls as well as individuals towards whom it was made, thus, we excluded the invitation data from this colony. Thus, the data for invitation calls were pooled from five colonies. Initiation videos were also analysed to explore impact of tandem leader's experience and/or group size effect on invitation call. As relocation progresses, on the one hand, TL acquire the experience of multiple initiations while, on the other hand, followers waiting to be taken to new nest site may become more eager and hence we may expect an influence of this in invitation duration. This was tested at the level of colony for the effect of reduced number of followers for which successive transport calls were given successive ranks (i.e. first transport as rank 1, second transport as rank 2 and so on) and the correlation between invitation duration and number of transport items (i.e. followers + brood items which are carried by TL) present at the old nest. The latter is counted *post facto* from the total number of transport events. In the generalized linear mixed model (GLMM) analysis to study the effect of various factors on invitation duration, the colony identity and transporter identity (nested within the colony identity) were considered as random effects. This allowed us to measure the effect of individual and colony level variation on the time taken for invitation. The type of transport and the number of items waiting to be transported were treated as fixed effects to analyse the dependence of invitation duration on these parameters. Further, to examine the effect of calling experience of that individual tandem leader, we sorted TL who performed over seven or more transports (*n* = 41) across five colonies. The attributes of the first and last invitation calls such as number of calls, number of receivers towards whom these calls were made and invitation duration, were compared.

### 2.2. Path

In order to elucidate the path taken during tandem running from the old nest to the new nest, we examined each transport event separately. The rectangular arena gave us an opportunity to test if *D. indicum* transporters preferred to walk along the edge and if so their preference towards the corner that was closer to their old nest when compared with the corner that was further away. To investigate, transporter's path preference from the old nest to the new nest (which was placed at the diagonally opposite corner) within the rectangular arena, the paths were pooled into three different categories. Short edge followed by long edge (SL path): if transporter first heads towards shorter edge followed by longer edge, diagonal route (D path): if they took a diagonal route, or long edge followed by short edge (LS path): if transporter first heads towards the longer edge followed by the shorter edge. It is important to note that the distance travelled along the SL and LS path will be comparable, while the D path is expected to be shorter.

Temporal dynamics of tandem running path was studied by following the fate of tandem leader and follower separately using video recordings of the path (electronic supplementary material, media S2). As two ants are involved in each tandem run, we randomly followed the actions of only one individual in a tandem run. While decoding the recordings, a coin was tossed to randomly decide whether to delineate the actions of a tandem leader or a follower, at the start of each tandem run. If the tandem leader's action was delineated in a given tandem run, the follower's action was delineated in the next tandem run that started at the old nest. In this manner, a minimum of 30% of total transport events from each of the six colonies were sampled. A total of 143 tandem runs were thus observed to characterize the path (of which 67 leaders and 76 followers were characterized). This protocol of delineating the actions of the leaders and followers separately enables us to sample these two categories independently and in a more robust manner. During tandem running, a tandem pair may encounter interruptions. Following this interruption, TL of *D*. *indicum* were seen to perform a sequence of behaviours ‘pause-search’ as documented for *Temnothorax albipennis* species [25]. The four possible outcomes that were observed following an interruption have been listed below:

1. The tandem leader pauses for a while and follower resumes contact and the pair continue towards the destination.
2. After a momentary pause, the tandem leader turns around and starts searching for the follower before resuming contact and continuing to the destination.
3. The tandem leader fails to find the follower subsequent to the ‘pause and search’, and returns back to the old nest. The leader may initiate another tandem run from the old nest.
4. During the pause or search behaviour, the tandem leader encounters a third ant (different from the initial follower) and resumes tandem running with the new follower to the destination.

In the outcomes 1 and 2, the pair walks successfully to the destination while in outcome 3, tandem leader returns to old nest and hence we consider such a walk as an unsuccessful walk. The outcome 4 is further studied to inspect the fidelity of tandem leader and followers during a tandem run. Hence, the interruption data analysed in this study characterize only the interruptions leading to outcome number 4. Hence, irrespective of whether we followed a tandem leader or a follower, we followed events that led to the separation of a tandem pair. Further, if we were focusing on the tandem leader then we recorded whether the tandem leader continues its tandem run with the same/another follower, after an interruption. Similarly, if we were focusing on the follower then we recorded whether the follower resumes tandem run with the same/alternative tandem leader. Thus, the number of times a partner switch occurred and whether the tandem run terminated at the new nest or not was recorded and analysed.

The follower was said to be lost after an interruption if she wandered around for more than 5 min. Similarly, path videos were analysed for BT by TL, where a transporter carried brood (pupa or big larva) to the new nest. The speed of individual transport event was documented. The distance covered divided by the time taken for a transport event was termed as its speed. Computation of speed was carried out by analysing the trajectory of the transporter through video records using custom routines written in Matlab. The thorax of each transporter was tracked every 0.04 s by marking the respective pixel. As the distance between the centres of adjacent pixels was 1.25 mm and the length of an ant was about 1 cm, a high level of precision was achieved. Every frame in each of the tracks was manually verified and then a five running average of the coordinates was performed to smoothen out the fluctuations. Transport of pupae by individual transporters provided the speed of BT. Also, transport of pupae by followers of tandem runs provided the speed of TRB. Similarly, tandem runs without any discernable brood were tracked to obtain the speed of a TR. To put these transport speeds into perspective, we required the average speed of an unencumbered tandem leader, i.e. the speed of returning TL (RL). Further, we examined the path efficiency of different transport categories. Path efficiency is defined as the ratio of displacement to distance covered. Thus, values close to 100% would imply that ants are taking short and less tortuous path between the two nests, while values closer to 0% would imply that the ants deviate from the shortest path and cover large distances to reach the target.

### 2.3. Termination

There were two possible termination points at the new nest, i.e. tunnel or chamber. Six different behaviours led to the termination of tandem runs. These behaviours are listed below: (i) ant interruption—tandem run termination due to interruption caused by other colony members getting in the way of the tandem pair or antennating either the leader or follower several times, (ii) non-ant interruptions—interruptions caused by physical factors such as pebbles or wind, (iii) stand still—tandem leader stops walking and freezes in one position causing the follower to disengage from the tandem leader and start other activities like exploring the nest, (iv) U-turn—tandem leader quickly takes a U-turn and walks away leaving the follower who is unable to catch up with the leader, (v) speed up—tandem leader abruptly increases her speed and loses contact with follower, and as a consequence the latter is unable to locate the tandem leader and thus starts exploring (vi) tandem leader–follower interaction—tandem leader turns and antennates follower and terminates the transport event, leading to separation of tandem pair. For each tandem run (with and without brood) and BT event, the time of tandem pair's or transporter's entry (*t*_e_), termination location (tunnel or chamber), time of tandem pair separation or dropping the brood (*t*_t_) and the cause of termination were recorded in order to analyse the termination (see electronic supplementary material, media S3). Termination time was hence calculated from the records (termination time inside nest = *t*_t _− *t*_e)._ While performing GLMM to analyse the effect of various factors on termination duration, colony identity and transporter identity (nested within the colony identity) were considered as random effects. The type of transport (BT, TR or TRB) was treated as a fixed effect.

Unless otherwise mentioned, the median and quartiles of the different parameters are reported. The ‘lme4’ package [26] in R Studio [27] was used to perform generalized linear mixed-effects model (GLMM) analysis. Two-tailed *p*-values less than or equal to 0.05 were considered significantly different. Tracking of ants for the above-mentioned analysis was performed using Matlab v. 7.12.0 (The Mathworks Inc. 2011).

## 3. Results

### 3.1. Initiation

**Figure 1.**
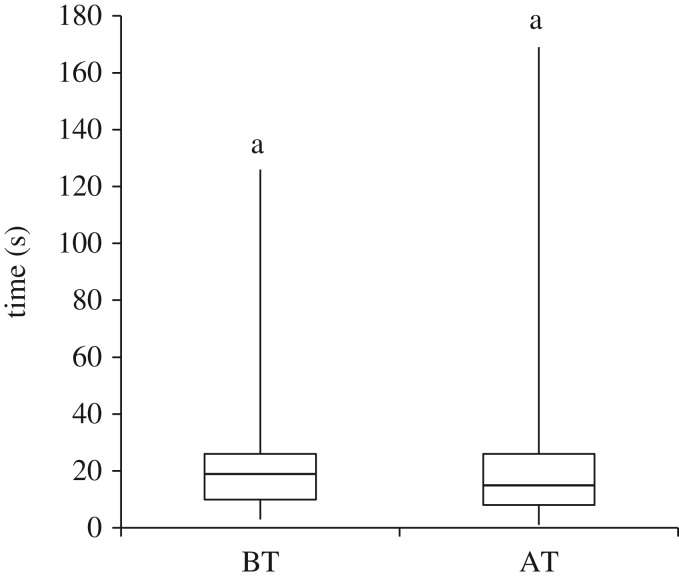
Invitation time: box plot representing time taken by transporters to initiate a brood transport (BT) and invitation time of tandem run with a follower (AT: adult transport). Line within the box represents the median and the box indicates the 25th and 75th percentiles. Whiskers extend to the minimum and the maximum data points. Same letter represents no significant difference between categories *p* > 0.05.

A total of 621 invitation calls were analysed from five colonies to characterize initiation of tandem running. Of these invitations 92% were successful in initiating either a tandem run or a BT. Data from the 572 successful invitations showed that TL made four to five calls (median, quartiles two and eight calls) towards three individuals (median, quartiles one and five individuals) and took 16 s (median, quartiles 9 and 26 s) to initiate a successful transport. Even newly eclosed (callow) ants were seen tandem running with leaders to the new nest. The type of transport did not influence the invitation time (GLMM, *p* = 0.15, electronic supplementary material, table S1*a*; figure 1). The colony identity and the transporter identity did not seem to have a prominent effect on the observed results. However, the number of transport items (brood + ants) remaining to be transported had a weak yet significant negative effect on the time taken to initiate a transport event (GLMM, *p* < 0.01; electronic supplementary material, table S1*a*). Further, among TL we observed no significant effect of experience when we compared the attributes like number of calls and number of receivers (GLMM, number of calls: *p* = 0.75 and number of receivers: *p* = 0.28, electronic supplementary material, table S1*b*). There was a significant increase in the invitation duration of the last call (20 s median, quartiles 9.5 and 34 s) in comparison to the invitation duration of the first call (14 s median, quartiles 9 and 21.5 s) by TL who performed a minimum of 7 tandem runs (GLMM, *p* < 0.01, electronic supplementary material, table S1*b*). By the end of the relocation, data collected from the initiation site show that 17.9% (median, quartiles 16.0 and 20.5%) of the colony became leaders and performed at least one tandem run. Across the five colonies studied, 77.8% (median, quartiles 70.5 and 80.3%) of the nest-mates became followers and were tandem run to the new nest. After being tandem run to the new nest, the proportion of these followers that participated in the relocation process as TL was 0.23 (median, quartiles 0.13 and 0.30). Further, the proportion of leaders who followed other leaders in tandem runs was 0.23 (median, quartiles 0.20 and 0.30).

### 3.2. Path

A total of 243 tandem run paths were examined and 217 (89.0%) of them reached the new nest successfully. In the other cases, either the tandem leader or follower returned back to the old nest. Across all relocations not a single follower was lost, as within 5 min following an interruption they resumed tandem running with another tandem leader.

Most of the interrupted tandem runs resumed with an alternative follower or leader (termed as switched partner) as the case be and the transport terminated at the new nest. The number of interruptions faced by TL (23.9%) and followers (43.4%) were not significantly different (GLMM, *p* = 0.16, electronic supplementary material, table S2*a*). However, in the 23.9% of tandem runs that were interrupted from the leader's point of view, the TL resumed with 1 (median, quartiles 0 and 1) followers. This was significantly smaller when compared with the followers who resumed tandem runs with 2 (median, quartiles 1 and 2) switched leaders (GLMM, *p* < 0.01, electronic supplementary material, table S2*b*), indicating that once followers faced an interruption they needed guidance from more than one leader to reach the new nest. Percentage of tandem runs in which the followers were holding brood (38.9%) that faced interruptions were comparable to percentage of tandem runs in which the follower was not holding any brood (44.8%, GLMM, *p* = 0.86, electronic supplementary material, table S2*a*). The path preference showed that the majority of the transports occur along the SL path both in the case of tandem running (73%) as well as BT (69%).

**Figure 2.**
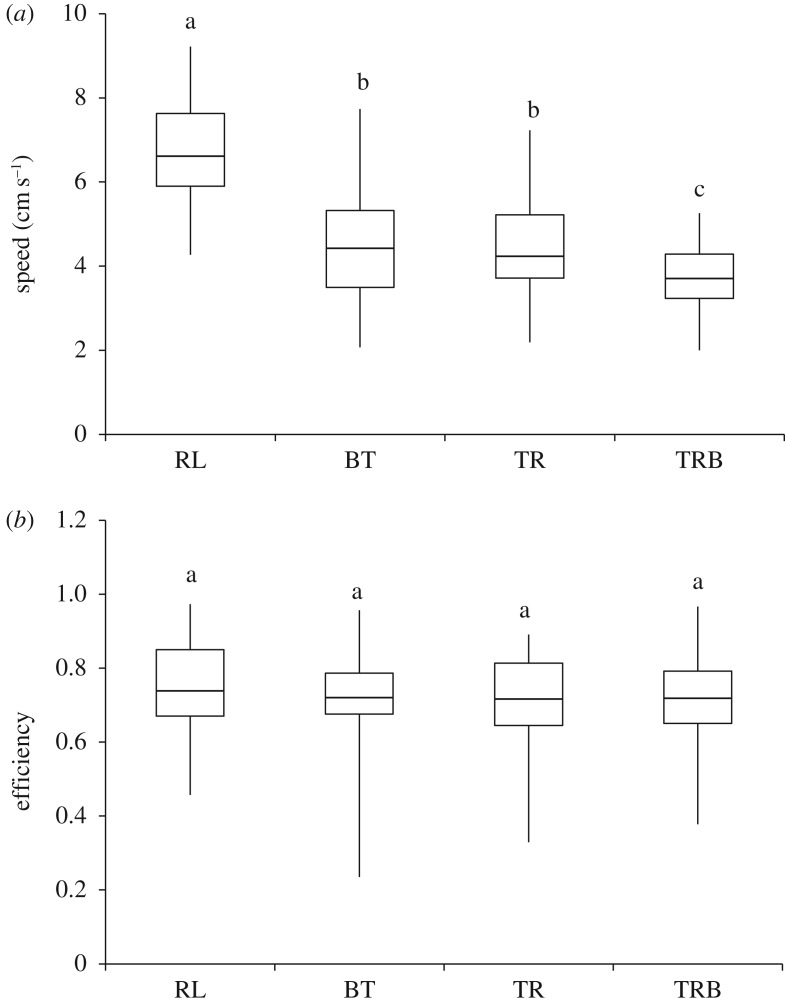
Speed and efficiency of transports: box plot representing (*a*) the speed and (*b*) the path efficiency (ratio of displacement to distance) of returning leaders (RL), brood transports (BT), tandem runs (TR) and tandem runs with follower carrying pupa (TRB). Line within the box represents the median and the box indicates the 25th and 75th percentiles. Whiskers extend to the minimum and the maximum data points. Same letter represents no significant difference between categories *p* > 0.05.

**Figure 3.**
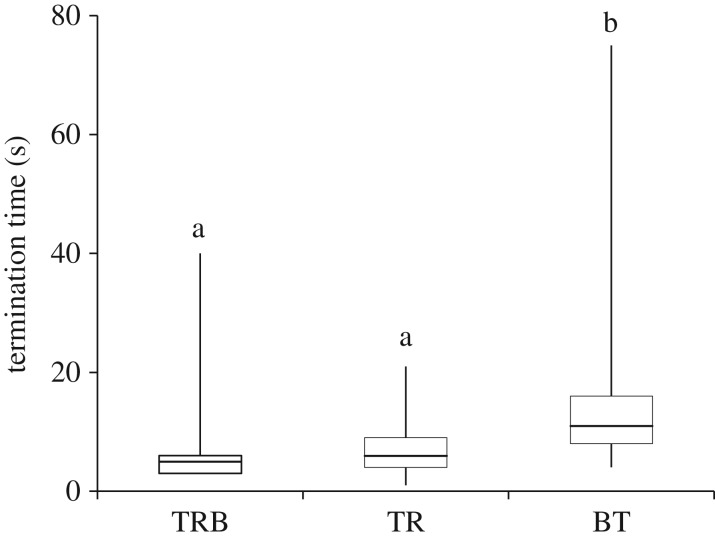
Temporal dynamics of termination: box plot representing time taken to terminate tandem runs with brood (TRB), tandem runs where the follower is not carrying any brood (TR) and brood transport (BT). Line within the box represents the median and the box indicates the 25th and 75th percentiles. Whiskers extend to the minimum and the maximum data points. Same letter represents no significant difference between categories *p* > 0.05.

Data from representative studies, on other ant species that carry, tandem run or use chemical trails in order to recruit, were compiled to get information regarding their speeds. The range of speed achieved as measured in body lengths per second (bl s^−1^) was from 0.38 to 7.50 for carrying, from 0.60 to 3.50 for tandem running and from 0.50 to 8.67 for walking (table 1). On measuring the speeds in the context of colony relocation in *D. indicum* (body length 1 cm), we found that the average speed of a returning leader (RL) was 6.6 cm s^−1^ (median, quartiles 5.9 and 7.6 cm s^−1^). This was significantly higher than both the speed for (BT) brood transport (median 4.4 cm s^−1^, quartiles 3.5 and 5.3 cm s^−1^, GLMM, *p* < 0.01, electronic supplementary material, table S2*c*; figure 2) and the speed for (TR) tandem run without brood (median 4.2 cm s^−1^, quartiles 3.7 and 5.2 cm s^−1^; GLMM, *p* < 0.01; electronic supplementary material, table S2*c*). However the speed for BT was comparable to that for TR (GLMM, *p* = 0.54). Further, the transport speed for (TRB) tandem run with follower carrying pupa (median 3.7 cm s^−1^, quartiles 3.2 and 4.3 cm s^−1^) was significantly lower than BT (GLMM, *p* < 0.01, electronic supplementary material, table S2*c*) as well as TR (GLMM, *p* < 0.01, electronic supplementary material, table S2*c*). The colony identity seemed to affect the observed speeds. Interestingly, the path efficiency (computed by the ratio of displacement to distance) of all these transports were greater than 70%. The path efficiency of BT (median 72.1% quartiles 67.6 and 78.7%), TR (median 71.7%, quartiles 64.5 and 81.3%) and TRB (median 71.9%, quartiles 65.1 and 79.2%) were comparable to the efficiency of RL (median 73.9%, quartiles 67.0 and 85.0%) as well as to each other (GLMM, *p* > 0.05, see electronic supplementary material, table S2*d*, for pairwise comparisons). This shows that the presence of a follower or brood in a transport event hinders the speed of a transporter by about 30%, but does not significantly affect the path efficiency (figure 3).

**Table 1.** Carrying and tandem running speed: a representative compilation of carrying and tandem running speeds of different species of ants available in the literature. All values have been normalized to their respective body lengths per second and the individual walking speeds of these ants have been presented. n.a. denotes that values are either not applicable or not available.

Sl. no.	species	mode of recruitment	body length^a^ (cm)	walking (bl s^−1^)	carrying (bl s^−1^)	TR (bl s^−1^)	reference
1	*Atta columbica*	chemical trail	0.6	8.67	7.50	n.a.	[28]
2	*Atta vollenweideri*	chemical trail	1.4	2.50	1.36	n.a.	[29]
3	*Leptogenys nitida*	chemical trail	0.45	6.44	0.76	n.a.	[30]
4	*Leptogenys processionalis*	chemical trail	0.7	6.09	5.84	n.a.	[31]
5	*Myrmecia pyriformis*	chemical trail	2	0.50	0.38	n.a.	[32]
6	*Pogonomyrmex rugosus*	chemical trail	0.8	5.63	2.81	n.a.	[33]
7	*Camponotus consobrinus*	tandem running	0.8	7.00	n.a.	3.50	[34]
8	*Temnothorax albipennis*	tandem running	0.25	3.36	1.84	0.60	[35]
9	*Diacamma indicum*	tandem running	1	6.60	4.50	4.35	

^a^AntWeb. Available from http://www.antweb.org. Accessed 23 September 2015.

### 3.3. Termination

In total, 228 terminations were analysed in this study including 69 tandem runs without brood, 92 tandem runs with the follower holding brood and 67 BT events by TL. The majority of tandem run terminations were caused as a result of interruption by other ants (88.2%) rather than any stereotypic behaviour. Percentage of terminations that occurred at the tunnel was 59.9% while the remaining terminated inside the new nest. Brood transporters did not hand over the brood items they were carrying to other workers after entering the new nest but placed them on the floor of the new nest. The termination dynamics was minimally impacted by colony and transporter identity. However, the type of transport has a significant effect on the time taken for termination of the transport event (GLMM, *p* < 0.01; electronic supplementary material, table S3*a*). The duration of termination of followers carrying brood (median 5 s, quartiles 3 and 8 s) and without brood (median 6 s, quartiles 4 and 9 s) were comparable and they were significantly lower than the termination duration of BT by transporters (median 11 s, quartiles 8 and 16 s, GLMM: TR-TRB: *p* = 0.67; TR-BT: *p* < 0.01; TRB-BT: *p* < 0.01; electronic supplementary material, table S3*a*). Significantly, a lower percentage (30.3%) of transport in which brood was carried by transporters (BT) terminated in the tunnel when compared with TR (71.0%) or TRB (73.6%, GLMM: TRB-BT, *p* < 0.01; TR-BT, *p* < 0.01; electronic supplementary material, table S3*b*).

## 4. Discussion

Recruitment of colony members for different purposes like resource utilization and colony defence is central to the success of social insects. In order to recruit to the right site at the appropriate time, these insects have evolved multiple methods of transferring information among themselves. Waggle dance in honeybees and chemical trail in many ant species serve as excellent examples [2,4]. Another such recruitment method is tandem running where two colony members walk in tandem from one location to other. In the model system used in this study, *D. indicum* employs this method to relocate all their colony members from one site to another. Tandem running is considered to be a primitive behaviour for the following three reasons: (i) the simplicity and slower rate of recruitment, (ii) its dependence on few individuals (TL) who possess the knowledge of new site(s) and (iii) the lack of reinforce-able chemical trails [8,12,36,37], but see [10,38]. Even though the occurrences of tandem running behaviour has been documented in several ant genera like *Cardiocondyla* [11], *Camponotus* [12], *Temnothorax* [5], *Harpagoxenus* [36], *Pachycondyla* [39] and many other [2,10], a detailed quantitative analysis of this behaviour, especially in a species that uses only this behaviour to recruit has not been conducted to the best of our knowledge.

In this study, we characterize this recruitment method by examining three different aspects: initiation, path and termination of tandem running. Leaders typically invite multiple potential followers rather than concentrate on a particular individual. With increasing experience of conducting tandem runs and thus improving their skills, leaders do not seem to improve their ability to make followers accept their invitation. As a next step, the differences between leaders within a relocation and their experience across different relocations would be required to fully appreciate their tandem running abilities in general and invitation behaviour in particular. The dynamics of relocation as it progresses changes the number of colony members present at the old nest, which is the site of tandem run initiation. As the exposed colony faces increased stress with passing time and the number of colony members present at the old nest decreases, it was expected that leader's invitation would be answered more eagerly by potential followers. However, this was not the case, instead there was a weak increase in the invitation duration as the relocation progressed. This could be due to the inter-individual variation in the response of followers. Even the newly eclosed callow were observed following TL indicating that accepting an invitation to become a follower and following is perhaps an innate behaviour. About one-fifth of the colony became leaders and more than three-quarters of the colony members followed a tandem leader to reach the new nest. One-quarter of the leaders followed other leaders in tandem runs to the new nest. Of the followers, one-quarter became leaders only after they were tandem run to the new nest. A comparison of the contribution of these individuals and the first-order leaders towards the relocation process requires further examination. Previous studies on this species in the natural habitat in the presence of multiple target sites [8] suggest that these leader following leader events play an important role in determining the dynamics of the colony relocation process.

Interruptions would cause tandem leader and follower to separate and their subsequent action would be mutually dependent. In order to avoid this, *a priori* random choice was made to track either the leader or the follower in a tandem run. This ensures that the information we collect about the success of tandem runs is independent for each of the participants. Note that our protocol did not require us to follow every tandem run performed during the relocation but a random subset of tandem runs performed by leaders and followers separately. Thus, all tandem runs performed by leaders do not add up to the same number of tandem runs performed by followers. Once a leader's tandem run is interrupted, she resumes it with another follower and leads this switched partner to the new nest. However, once a follower's tandem run is interrupted she tends to get lost a second time and generally requires two leaders to show her to the new nest. Interestingly, none of the followers in the entire study got lost. This is because most of the tandem runs occurred in one general region of this rectangular sand arena—the short edge followed by the long edge. Thus, the plethora of leaders walking to and from the nests encountered any lost follower within the stipulated 5 min observation window. While the advantages of having a relatively common path are clear, it has to be pointed out that leaders show the remarkable ability to assess the situation and take up the required call. For an insect, the size of these ants with a small brain, these returning leaders show a clear conditional response. Having started their trip back to the old nest, they stop midway on encountering a potential follower. They invite this member and upon being accepted turn 180° and head off in the opposite direction towards the new nest.

The number of interruptions faced and the path efficiency of followers holding brood and those that do not hold brood are comparable. Followers holding brood as they tandem run behind a leader are in fact significantly slower than transporters who carry the brood by themselves. Analysis of the speed of these transports reveal that (for a displacement of 1 m) transportation of one follower by tandem running her and one pupa by BT and two return walks that the TL would be making altogether would on average take 102 s. While transportation of the same through a coupled adult-brood event, i.e. a leader tandem running a follower who is holding a pupa in her mandibles and one return walk that the leader would have to make would take 57 s on average. Here, we see that coupled adult-BT, though slower than the unencumbered tandem run, is economical for the colony as it contributes to the transport of two items in a single transport event. This in turn saves energy as well as time invested for a colony relocation process. This increased efficiency of coupled transport is perhaps the reason why the majority of the colony's brood is transported in this manner in *D. indicum* [40].

To comprehend tandem running in *D. indicum*, it will be meaningful to compare the same with other ant species that perform recruitment via chemical trails and tandem running. A representative compilation of the information available in the literature is presented in table 1. *Diacamma indicum* being a monomorphic ant with a body length of 1 cm, returning leaders covered more than six times their body length per second (bl s^−1^). The distance covered is presented in body lengths for ease of comparison with other species having different sizes. While a *D. indicum* transporter carried a brood item, its speed reduced by 30%. Studies in different contexts across different species around the world show that carrying a load of about 1 : 1 body weight causes 10% to over 85% reduction in their speeds (table 1). In the few tandem running species where such studies have been conducted [6,34,35], tandem runs were 50 to over 80% slower than an individual ant. Though a significant reduction in speed was observed in *D. indicum* ants, tandem runs in this species were only about 34% slower than an individual ant. In addition, the path efficiency for tandem runs and number of interruptions were comparable to the unencumbered walks by the corresponding leaders. This together signifies the efficiency of tandem running as a mode of recruitment employed by these ant colonies. Even though tandem running is considered primitive, this strategy is comparable in terms of speed to other more evolved methods like chemical trails.

A detailed study of laboratory-based relocations on *T. curvispinosus*, a species that uses tandem running only in the initial phase of colony relocation and switches to carrying both adults and brood to the new nest, found that tandem running was three times slower than carrying and about two-thirds of tandem runs were interrupted before reaching the new nest [19]. Comparing tandem running in *D. indicum*, *T. albipennis* and *T. curvispinosus*, we find that *D. indicum* achieves higher speed (about 4.2 bl s^−1^) and faces less interruptions (about 34%) during tandem runs as documented in laboratory studies. As tandem running is the only recruitment method used by *D. indicum*, they have not only coupled BT with tandem running but have perhaps also improved their efficiency of performing it. It may, however, be reiterated that inter-individual/colony variability as well as factors like colony size, number of scouts involved in the process, the distance over which they travel and the nature of the terrain will influence the overall transport dynamics.

Unlike initiation, no stereotyped termination behaviour was observed and the majority of tandem runs terminated in the tunnel after being interrupted by other ants present inside the tunnel or chamber. Ant interruption causing tandem run termination was similar to ant interruptions observed during path, i.e. while travelling from old to new nest. Interestingly, two very different responses were observed from the TL towards the caused interruption. Once a tandem pair reaches the new nest, TL paid no attention to the otherwise lost follower. She turned and/or groomed herself or vice versa, and exited from the new nest. By contrast, if the same interruption happens during the path, leaders wait and later search for follower. Termination dynamics of tandem run differs from BT both in terms of site of transport and time taken to terminate. The latter took longer and most of it terminated inside the chamber, even when colony members were present inside the tunnel. Also, TL were seen to keep brood on the ground but only after some unknown considerations which took additional time. They walked around holding the brood and seemed to examine the new nest, with their antennae, several times before placing it inside. This adds to the efficiency gained by coupling brood and adult transport because if TL start relocating all the brood items, they would take similar time to initiate and carry but double the time to terminate the BT and become available for the next transport event. This would impact the overall time required for relocation of the colony, which in turn would increase the risk of the colony's exposure to harsh environment and predators.

The conditional response of TL to interruptions, the lack of fidelity to tandem partners, the increased efficiency achieved by coupled adult-BT in the background of speed comparable to other recruitment methods makes tandem running an efficient mode of recruitment in *D. indicum*. Based on the current findings, we postulate that this primitive recruitment behaviour is just another means to achieve the same end. Further insight requires inter-species studies preferably conducted in their natural habitat to fully appreciate these recruitment methods when employed exclusively or in different combinations.

## Supplementary Material

Sample video of the three components of tandem running; initiation, path and termination. Three Tables regarding the details of the results from GLMM analysis

## Supplementary Material

Supplementary Media 1

## Supplementary Material

Supplementary Media 2

## Supplementary Material

Supplementary Media 3

## Data accessibility

Data files are available at the Dryad Digital Repository: http://dx.doi.org/10.5061/dryad.n4h17 [41].
