# Supplementary material for: Characterization of recruitment through tandem running in an Indian queenless ant Diacamma indicum
Source: R Soc Open Sci. 2017 Jan 18;4(1):160476. doi: 10.1098/rsos.160476 (PMC5319314; doi:10.1098/rsos.160476)
Supplement: Sample video of the three components of tandem running; initiation, path and termination. Three Tables regarding the details of the results from GLMM analysis [file rsos160476supp1.doc]

**Characterization of recruitment through tandem running in an Indian queenless ant *Diacamma indicum***

**Supplementary Files**

**Rajbir Kaur^1,2^, Joby Joseph^3^, Karunakaran Anoop^1^ and Annagiri Sumana^1*^**

*Address for correspondence:

^1^Behaviour & Ecology Lab, Department of Biological Sciences

Indian Institute of Science Education and Research, Kolkata

Mohanpur – 741246, India

^2^Institute of Zoology,

Johannes Gutenberg University of Mainz,

Johannes von Müller Weg 6, 55099 Mainz, Germany

^3^Center for Neural & Cognitive Sciences

University of Hyderabad, Hyderabad-500 046, India

^*^Email: sumana@iiserkol.ac.in

# Phone: + 91-(0)33-25873017; Fax: + 91-(0)33-25873028

**Supplementary Media 1**

Media depicting the activity at the old nest during the progress of a relocation. A transporter (marked Yellow Yellow Yellow) is observed to successfully initiate tandem run with a follower (marked Blue Blue Blank).

**Supplementary Media 2**

Media depicting the activity at the experimental arena during the progress of a relocation. Multiple transporters are seen performing tandem runs as well as brood transports from the old nest (bottom right) to the new nest (attached through tunnel at the top left).

**Supplementary Media 3**

Media depicting the termination of a tandem run at the new nest. The leader (marked Orange Blank Blank) leads a follower (marked Blank White Blank) to the new nest. Following termination, the leader may return back to initiate subsequent transports

**Supplementary Table 1a**

Results of the GLMM analysis considering a Gamma distribution to study the effect of type of transport as well as number of ants and brood remaining to be transported, on the invitation duration of tandem leaders (*variables description mentioned at the end of document).

| **Response variable** | | | | |
| --- | --- | --- | --- | --- |
| **Model** | glmer(duration_invitation ~ transport_type + number_ant_brood + (1|colony_ID/leader_ID), family="Gamma") | | | |
| **Fixed effects** | **Estimate** | **Std. Error** | **t value** | **Pr(>|z|)** |
| (Intercept) | 3.55E-02 | 5.62E-03 | 6.313 | **< 0.001** |
| transport_typeTR | 4.87E-03 | 3.36E-03 | 1.451 | 0.147 |
| number_ant_brood | 2.23E-04 | 3.59E-05 | 6.204 | **< 0.001** |
| **Random effects** | **Name** | **Variance** | **Std. Dev.** | |
| leader_ID:colony_ID | (Intercept) | 7.37E-05 | 0.008582 | |
| colony_ID | (Intercept) | 5.45E-05 | 0.007384 | |
| Residual |  | 5.87E-01 | 0.766433 | |
| Number of obs: 572, groups: leader_ID:colony_ID, 68; colony_ID, 5 | | | | |

**Supplementary Table 1b**

Results of the GLMM analysis considering a Binomial distribution to study the effect of leader experience on parameters like number of calls, number of receivers and duration of the call by tandem leaders (*variables description mentioned at the end of document).

| **Response variable** | | | | |
| --- | --- | --- | --- | --- |
| **Model** | glmer(call_number ~ number_calls * number_receivers + duration_invitaion + (1|colony_ID/leader_ID), family="binomial") | | | |
| **Fixed effects** | **Estimate** | **Std. Error** | **z value** | **Pr(>|z|)** |
| (Intercept) | -1.12687 | 6.42E-01 | -1.756 | 0.079 |
| number_calls | 4.03E-02 | 1.28E-01 | 0.315 | 0.753 |
| number_receivers | -2.05E-01 | 1.90E-01 | -1.076 | 0.282 |
| duration_invitaion | 0.12857 | 3.69E-02 | 3.481 | **< 0.001** |
| number_calls:number_receivers | -0.03093 | 1.51E-02 | -2.054 | **0.040** |
| **Random effects** | **Name** | **Variance** | **Std. Dev.** | |
| leader_ID:colony_ID | (Intercept) | 9.51E-17 | 9.75E-09 | |
| colony_ID | (Intercept) | 0.00E+00 | 0.00E+00 | |
| Number of obs: 81, groups: leader_ID:colony_ID, 41; colony_ID, 5 | | | | |

**Supplementary Table 2a**

Results of the GLMM analysis considering a Binomial distribution to study the effect of type of transport as well as the ant (leader or follower) being observed, on tandem run interruption (*variables description mentioned at the end of document).

| **Response variable** | | | | |
| --- | --- | --- | --- | --- |
| **Model** | glmer(interruption ~ ant_type + transport_type + (1|colony_ID), family="binomial") | | | |
| **Fixed effects** | **Estimate** | **Std. Error** | **z value** | **Pr(>|z|)** |
| (Intercept) | -6.92E-02 | 3.24E-01 | -0.214 | 0.831 |
| ant_typeTL | -4.64E-01 | 3.30E-01 | -1.406 | 0.160 |
| transport_typeTRB | -6.80E-02 | 3.86E-01 | -0.176 | 0.860 |
| **Random effects** | **Name** | **Variance** | **Std. Dev.** | |
| colony_ID | (Intercept) | 2.44E-01 | 0.4938 | |
| Number of obs: 163, groups: colony_ID, 6 | | | | |

**Supplementary Table 2b**

**Results of the GLMM analysis considering a Poisson distribution to study the effect of ant (leader or follower) being observed on the number of partner switches, following an interrupted tandem run (*variables description mentioned at the end of document).**

| **Response variable** | | | | |
| --- | --- | --- | --- | --- |
| **Model** | glmer(switch ~ ant_type + (1|colony_ID), family="poisson") | | | |
| **Fixed effects** | **Estimate** | **Std. Error** | **z value** | **Pr(>|z|)** |
| (Intercept) | 4.44E-01 | 1.83E-01 | 2.425 | **0.015** |
| ant_typeTL | -9.87E-01 | 2.70E-01 | -3.652 | **< 0.001** |
| **Random effects** | **Name** | **Variance** | **Std. Dev.** | |
| colony_ID | (Intercept) | 8.02E-02 | 0.2832 | |
| Number of obs: 67, groups: colony_ID, 6 | | | | |

**Supplementary Table 2c**

Results of the GLMM analysis considering a Gamma distribution to study the effect of type of transport on the speed of transporters (*variables description mentioned at the end of document).

| **Response variable** | | | | |
| --- | --- | --- | --- | --- |
| **Model** | glmer(speed ~ transport_type + (1|colony_ID), family= "Gamma") | | | |
| **Fixed effects** | **Estimate** | **Std. Error** | **t value** | **Pr(>|z|)** |
| (Intercept) | 2.26E+01 | 9.87E-01 | 22.866 | **< 0.001** |
| transport_typeRL | -7.48E+00 | 8.83E-01 | -8.47 | **< 0.001** |
| transport_typeTR | 6.36E-01 | 1.03E+00 | 0.618 | 0.537 |
| transport_typeTRB | 4.29E+00 | 1.11E+00 | 3.874 | **< 0.001** |
| **Random effects** | **Name** | **Variance** | **Std. Dev.** | |
| colony_ID | (Intercept) | 7.61E-01 | 0.8725 | |
| Residual |  | 5.13E-02 | 0.2266 | |
| Number of obs: 207, groups: colony_ID, 6 | | | | |
| **Test contrasts of factor interactions** | | | | |
| Chisq Test: | P-value adjustment method: holm | | | |
|  | **Value** | **Df** | **Chisq** | **Pr(>Chisq)** |
| BT-RL | 0.13369 | 1 | 71.7361 | **< 0.001** |
| BT-TR | -1.57181 | 1 | 0.3816 | 0.537 |
| BT-TRB | -0.23289 | 1 | 15.0079 | **< 0.001** |
| RL-TR | -0.12321 | 1 | 82.3088 | **< 0.001** |
| RL-TRB | -0.08493 | 1 | 145.6095 | **< 0.001** |
| TR-TRB | -0.27339 | 1 | 10.6269 | **0.002** |

**Supplementary Table 2d**

Results of the GLMM analysis considering a Gamma distribution to study the effect of type of transport on the path efficiency of transporters (*variables description mentioned at the end of document).

| **Response variable** | | | | |
| --- | --- | --- | --- | --- |
| **Model** | glmer(efficiency ~ transport_type + (1|colony_ID), family= "Gamma") | | | |
| **Fixed effects** | **Estimate** | **Std. Error** | **t value** | **Pr(>|z|)** |
| (Intercept) | 1.39E+00 | 5.58E-02 | 24.91 | **< 0.001** |
| transport_typeRL | -4.64E-02 | 4.59E-02 | -1.01 | 0.312 |
| transport_typeTR | 1.94E-03 | 4.59E-02 | 0.042 | 0.966 |
| transport_typeTRB | 1.83E-02 | 4.59E-02 | 0.399 | 0.690 |
| **Random effects** | **Name** | **Variance** | **Std. Dev.** | |
| colony_ID | (Intercept) | 2.66E-03 | 0.05156 | |
| Residual |  | 2.38E-02 | 0.15418 | |
| Number of obs: 207, groups: colony_ID, 6 | | | | |
| **Test contrasts of factor interactions** | | | | |
| Chisq Test: | P-value adjustment method: holm | | | |
|  | **Value** | **Df** | **Chisq** | **Pr(>Chisq)** |
| BT-RL | 21.56 | 1 | 1.0201 | 1 |
| BT-TR | -515.74 | 1 | 0.0018 | 1 |
| BT-TRB | -54.6 | 1 | 0.1596 | 1 |
| RL-TR | -20.69 | 1 | 1.1214 | 1 |
| RL-TRB | -15.45 | 1 | 2.0645 | 0.905 |
| TR-TRB | -61.06 | 1 | 0.1277 | 1 |

**Supplementary Table 3a**

Results of the GLMM analysis considering a Gamma distribution to study the effect of type of transport on the termination duration of tandem leaders (*variables description mentioned at the end of document).

| **Response variable** | | | | |
| --- | --- | --- | --- | --- |
| **Model** | glmer(termination_duration ~ transport_type + (1|colony_ID/leader_ID), family= "Gamma") | | | |
| **Fixed effects** | **Estimate** | **Std. Error** | **t value** | **Pr(>|z|)** |
| (Intercept) | 8.59E-02 | 1.22E-02 | 7.052 | **< 0.001** |
| transport_typeTR | 7.46E-02 | 1.34E-02 | 5.588 | **< 0.001** |
| transport_typeTRB | 8.05E-02 | 1.21E-02 | 6.641 | **< 0.001** |
| **Random effects** | **Name** | **Variance** | **Std. Dev.** | |
| leader_ID:colony_ID | (Intercept) | 9.40E-04 | 0.03065 | |
| colony_ID | (Intercept) | 1.35E-04 | 0.01162 | |
| Residual |  | 3.80E-01 | 0.61675 | |
| Number of obs: 226, groups: leader_ID:colony_ID, 76; colony_ID, 6 | | | | |
| **Test contrasts of factor interactions** | | | | |
| Chisq Test: | P-value adjustment method: holm | | | |
|  | **Value** | **Df** | **Chisq** | **Pr(>Chisq)** |
| BT-TR | -13.405 | 1 | 31.2313 | **< 0.001** |
| BT-TRB | -12.418 | 1 | 44.1075 | **< 0.001** |
| TR-TRB | -168.747 | 1 | 0.1786 | 0.673 |

**Supplementary Table 3b**

Results of the GLMM analysis considering a Gamma distribution to study the effect of type of transport on the termination point of tandem leaders (*variables description mentioned at the end of document).

| **Response variable** | | | | |
| --- | --- | --- | --- | --- |
| **Model** | glmer(termination_point ~ transport_type + (1|colony_ID/leader_ID), family= "binomial") | | | |
| **Fixed effects** | **Estimate** | **Std. Error** | **t value** | **Pr(>|z|)** |
| (Intercept) | -1.99E-01 | 1.12E+00 | -0.178 | 0.859 |
| transport_typeTR | 2.37E+00 | 5.32E-01 | 4.449 | **< 0.001** |
| transport_typeTRB | 2.83E+00 | 5.36E-01 | 5.282 | **< 0.001** |
| **Random effects** | **Name** | **Variance** | **Std. Dev.** | |
| leader_ID:colony_ID | (Intercept) | 1.79E-01 | 0.4228 | |
| colony_ID | (Intercept) | 5.95E+00 | 2.4382 | |
| Number of obs: 226, groups: leader_ID:colony_ID, 76; colony_ID, 6 | | | | |
| **Test contrasts of factor interactions** | | | | |
| Chisq Test: | P-value adjustment method: holm | | | |
|  | **Value** | **Df** | **Chisq** | **Pr(>Chisq)** |
| BT-TR | 0.08559 | 1 | 19.7974 | **< 0.001** |
| BT-TRB | 0.05576 | 1 | 27.9024 | **< 0.001** |
| TR-TRB | 0.38686 | 1 | 1.1477 | 0.284 |

*Description of variables used in the models mentioned above

duration_invitation: time taken by a tandem leader to initiate a successful transport event

transport_type: tandem run (TR), tandem run with brood (TRB) and brood transport (BT)

number_ant_brood: number of transportable items at the initiation site

colony_ID: colony identity

leader_ID: tandem leader identity

call_number: binomial representing whether the invitation was the first (0) or the last (1) of the transport events sampled for the particular leader

number_calls: number of calls made towards fellow colony members in an effort to initiate a transport event

number_receivers: number of colony members towards which “number_call” were made

interruption: number of interruptions faced by the focal individual between start to stop point

ant_type: tandem leader or follower

transport_type: tandem run, tandem run with brood, brood transport

switch: number of times tandem running partner being exchanged by the focal individual “ant type”

speed: speed of transport event

efficiency: efficiency of the transport event

termination_duration: time taken to terminate a transport event inside the new nest

termination_point: tunnel and box, two locations inside the new nest where transport events may terminate
