# Supplementary figures and images for: Characterization of recruitment through tandem running in an Indian queenless ant Diacamma indicum
Source: R Soc Open Sci. 2017 Jan 18;4(1):160476. doi: 10.1098/rsos.160476 (PMC5319314; doi:10.1098/rsos.160476)

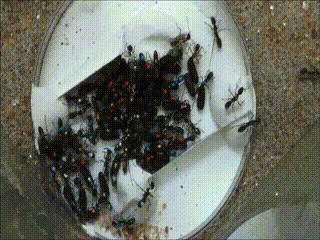

Supplement: Supplementary Media 1 [file rsos160476supp2.gif]

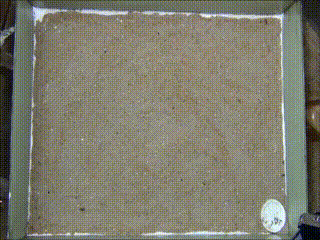

Supplement: Supplementary Media 2 [file rsos160476supp3.gif]

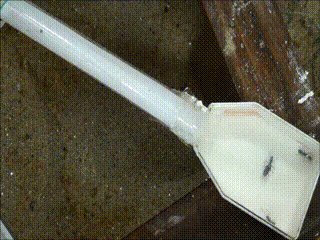

Supplement: Supplementary Media 3 [file rsos160476supp4.gif]
